# The Persistence of HIV Diversity, Transcription, and Nef Protein in Kaposi’s Sarcoma Tumors during Antiretroviral Therapy

**DOI:** 10.3390/v14122774

**Published:** 2022-12-13

**Authors:** David J. Nolan, Rebecca Rose, Rongzhen Zhang, Alan Leong, Gary B. Fogel, Larissa L. S. Scholte, Jeffrey M. Bethony, Paige Bracci, Susanna L. Lamers, Michael S. McGrath

**Affiliations:** 1 Bioinfoexperts, LLC, Thibodaux, LA 70301, USA; 2 Departments of Laboratory Medicine, Pathology and Medicine, The University of California at San Francisco, San Francisco, CA 94110, USA; 3 Natural Selection, Inc., San Diego, CA 92127, USA; 4 Department of Microbiology, Immunology and Tropical Medicine, The George Washington University, Washington, DC 20037, USA; 5 The AIDS and Cancer Specimen Resource, San Francisco, CA 94110, USA

**Keywords:** HIV Nef, viral reservoirs, Kaposi’s sarcoma, HIV transcription

## Abstract

Epidemic Kaposi’s sarcoma (KS), defined by co-infection with Human Herpes Virus 8 (HHV-8) and the Human Immunodeficiency Virus (HIV), is a major cause of mortality in sub-Saharan Africa. Antiretroviral therapy (ART) significantly reduces the risk of developing KS, and for those with KS, tumors frequently resolve with ART alone. However, for unknown reasons, a significant number of KS cases do not resolve and can progress to death. To explore how HIV responds to ART in the KS tumor microenvironment, we sequenced HIV *env-nef* found in DNA and RNA isolated from plasma, peripheral blood mononuclear cells, and tumor biopsies, before and after ART, in four Ugandan study participants who had unresponsive or progressive KS after 180–250 days of ART. We performed immunohistochemistry experiments to detect viral proteins in matched formalin-fixed tumor biopsies. Our sequencing results showed that HIV diversity and RNA expression in KS tumors are maintained after ART, despite undetectable plasma viral loads. The presence of spliced HIV transcripts in KS tumors after ART was consistent with a transcriptionally active viral reservoir. Immunohistochemistry staining found colocalization of HIV Nef protein and tissue-resident macrophages in the KS tumors. Overall, our results demonstrated that even after ART reduced plasma HIV viral load to undetectable levels and restored immune function, HIV in KS tumors continues to be transcriptionally and translationally active, which could influence tumor maintenance and progression.

## 1. Introduction

Epidemic Kaposi’s sarcoma (KS), defined by co-infection with Human Herpes Virus 8 (HHV-8) [1] and the Human Immunodeficiency Virus (HIV), displays aggressive clinical features [2] and is a major cause of mortality in sub-Saharan Africa. Antiretroviral therapy (ART) has significantly reduced the risk of developing KS, and in those with epidemic KS, tumors frequently resolve with ART [3]. However, a significant subset of people living with HIV (PLWH) on ART do not resolve KS and progress to death, despite improved CD4+ T-cell counts and low viral loads [3]. This is especially true in Sub-Saharan Africa where seroprevalence of both viruses is the highest worldwide [4,5] and chemotherapy as a second-line treatment may not be available or effective in reducing tumor burden [6,7]. Overall, the combination of factors that leads to persistent or progressive KS after ART initiation remains ill-defined, and pre-ART biomarkers for those at risk are still lacking [3,8].

Spindle cells, the defining abnormal elongated cells in KS tumors, which feature markers of endothelial and lymphatic origin, express high levels of vascular endothelial growth factor that promotes extensive vascularization within the tumor [9]. KS tumors are polyclonal and, unlike in classical cancer metastasis, individual tumors may arise independently [9]. KS tumors are histologically complex, involving interactions among HHV8-infected spindle cells with immune cells, including T cells, B cells, and macrophages, which may be HIV infected in epidemic KS cases [10,11,12]. The much higher frequency of KS in co-infected PLWH as compared to those only infected with HHV-8 suggests a role for HIV in KS pathogenesis.

HIV proteins Tat and Nef have synergistic effects with HHV-8 [13,14] and promote the oncogenesis of KS and other HIV-associated cancers [15,16,17,18]. HIV suppression of cytotoxic immune cell responses [19,20], along with HHV-8 host immune modulation strategies, could allow for progression of KS and the persistence of infected cells producing viral proteins in the tumor microenvironment, even after immune function is restored with ART. HIV Nef protein protects infected primary cells from cytotoxic T lymphocytes (CTLs) by downregulating major histocompatibility complex (MHC) class I presentation [21,22,23,24,25]. Nef allows infected cells to avoid natural killer (NK) cell detection through selective downregulation of human leukocyte antigen (HLA) presentation [26,27]. The influence of these viral proteins on the maintenance or progression of KS during ART remains unexplored.

Only a few studies have examined HIV diversity in cancer tissues derived from living subjects or autopsy specimens [28,29,30,31,32]. The work presented here leverages a collection of blood samples and cutaneous KS tumor punch biopsies in four participants that had unresponsive or progressive KS after 180–250 days of ART. To evaluate the presence, diversity, and expression of HIV in the participants before and after ART, we isolated DNA and RNA from plasma, peripheral blood mononuclear cells (PBMCs), and fresh-frozen tumor punch biopsies and performed single-genome sequencing experiments followed by phylogenetic analysis. To elucidate the presence of HIV Nef protein in infected cells of the KS tumors, we performed immunohistochemistry staining (IHC) experiments on matched formalin-fixed tumor biopsies.

## 2. Materials and Methods

### 2.1. Source of Specimens Derived from KS Subjects

The current study used tissues and blood specimens from subjects enrolled in the Antiretrovirals for Kaposi’s Sarcoma (ARKS) cohort [33] obtained through the AIDS and Cancer Specimen Resource (ACSR). The ACSR provided limited and de-identified demographic and clinical information on the four cases studied. The primary purpose of ARKS was to determine whether a protease-inhibitor (PI-based) ART regimen (lopinavir/ritonavir plus emtricitabine/tenofovir) was superior to a non-nucleoside reverse transcriptase inhibitor (NNRTI)-based ART regimen (efavirenz plus emtricitabine/tenofovir) in promoting the regression of KS tumor burden in persons with AIDS-related KS in sub-Saharan Africa. ARKS enrolled participants with AIDS-related KS in Kampala, Uganda, randomly assigned them to either a PI-based ART or an NNRTI-based ART regimen and observed them for one year to determine the response in their KS to therapy (N = 224). To be eligible for ARKS, subjects had to have histologically confirmed KS through skin punch biopsies and no history of prior ART [34,35]. No subjects were classified as having advanced KS (defined as an urgent indication for chemotherapy and/or functionally disabling complications of KS). Subjects also could not have evidence of current active untreated opportunistic infection (e.g., tuberculosis or cryptococcosis) or other malignancy. Longitudinal blood samples and tumor biopsies were collected from April 2007 to February 2011, prior to and during the drug regimen trial. In 95% of subjects, plasma HIV viral loads were undetectable by week 12 of ART. Within the first 12 months, approximately one-third of the subjects died or required chemotherapy because of progressive KS while taking ART (N = 70). Subjects with progressive KS had repeat biopsies while on ART. This progressive subgroup of the ARKS collection for which adequate samples were available to perform the sequencing and immunohistochemistry experiments in this study at baseline and after 180 or more days of ART prior to chemotherapy (N = 18) is represented by the 4 subjects in the current study.

### 2.2. RNA/DNA Extraction

Baseline and post-treatment tumor, PBMC and plasma specimens with ART administration longer than 180 days prior to initiation of chemotherapy were analyzed in this study. Cell-free plasma samples (200 µL) were processed to isolate viral RNA using the Viral Quick-RNA kit (Zymo #R1034), according to manufacturer’s instructions with a 14 µL elution volume. PBMC samples (~1 × 106) were processed to separately isolate total DNA and RNA using the AllPrep DNA/RNA/miRNA Universal Kit (Qiagen #80224, Hilden, Germany) following manufacturer’s guidance using a Qiashredder column (Qiagen #79656) for homogenization after lysis and a DNase I digestion on the RNeasy Mini spin column. The eluted RNA was concentrated using RNeasy MinElute Cleanup Kit (Qiagen #74204). Fresh-frozen tumor biopsy samples preserved in RNA later were cut into ~10 mg sections and each section homogenized in 2 mL RNase-free tubes containing equal proportions of 0.9–2.0 mm and 3.2 mm RNase-free SST beads using a BeadBug 6 Homogenizer (Benchmark Scientific D1036, Sayreville, NJ, USA) set for 4 cycles of 90 sec at 4350 RPM. Samples were chilled in an ice bath between cycles. The resulting lysate was further homogenized using a Qiashredder column (Qiagen #79656). The homogenized lysates were processed using the AllPrep DNA/RNA/miRNA Universal Kit (Qiagen #80224) following manufacturer’s guidance including a DNase I digestion on the RNeasy Mini spin column. The eluted RNA was concentrated using RNeasy MinElute Cleanup Kit (Qiagen #74204). Immediately after processing samples to extract RNA, 11 µL RNA was reverse-transcribed using the Invitrogen Superscript IV first-strand synthesis system (Thermo Fisher Scientific #18091050, Waltham, MA, USA), according to manufacturer’s recommendations with a 2:1 mixture of 50 ng/μL random hexamers: 50 µM Oligo d(T)20. Complementary DNA and genomic DNA were frozen at −200 °C until use. RNA and DNA concentrations were measured using the Invitrogen Qubit 3.0 fluorimeter (Thermo Fisher Scientific #Q33216), and RNA integrity was assessed using the Bioanalzyer 2100 (Agilent Technologies #G2939BA, Santa Clara, CA, USA). RNA and DNA extractions, cDNA synthesis, and first-round PCR setup were conducted in an amplicon-free biosafety cabinet using separate laboratory equipment where no amplified PCR products or recombinant plasmids were allowed, and work surfaces and equipment were thoroughly cleaned before and after use with Eliminase (Decon Labs, Inc., King of Prussia, PA, USA).

### 2.3. HIV Sequencing

A modified single-genome sequencing protocol was performed based on previously published methods [36]. The cDNA and genomic DNA were diluted serially until an average of 30% or less of the nested PCR reactions were positive. During the first-round PCR, diluted cDNA or genomic DNA was amplified in 25 µL reactions containing 1× Invitrogen Platinum™ II Hot-Start Green PCR Master Mix (Thermo Fisher Scientific #14001014) and 0.2 µM of each primer: VIF_F2, 5′-TGGAAAGGTGAAGGGGCAGTA-3′ and 3UTR_R1, 5′-TATTGAGGCTTAAGCAGTGGGTTC-3′ (4956–4976 and 9615–9592 of HIV-1 HXB2, respectively) with the following cycling parameters: initial denaturation 94 °C for 2 min, then 35 cycles of 94 °C for 15 s, 60 °C for 30 s, 68 °C for 3 min, followed by 68 °C for 10 min. Second-round nested PCR consisted of 2 µL of the first-round PCR added to a 25 µL reaction consisting of 1× Invitrogen Platinum™ II Hot-Start Green PCR Master Mix (Thermo Fisher Scientific #14001014) and 0.2 µM of each primer: ENV_F1, 5′-GGCTTAGGCATCTCCTATGGCAGGAAGAA-3′ and 3UTR_R6, 5′-GCTTATATGCAGCATCTGAGGG-3′ (5954–5982 and 9497–9518 of HIV-1 HXB2, respectively) with the following cycling parameters: initial denaturation 94 °C for 2 min, then 35 cycles of 94 °C for 15 s, 60 °C for 30 s, 68 °C for 3 min, followed by 68 °C for 10 min. Second-round PCRs were visualized on 1% agarose gels stained with GelRed Dye (Biotium #41003, Fremont, CA, USA), and reactions containing a single band were selected for sequencing. The primers specific to this publication were designed using Primer3 [37] and observing regions of conservation in alignments of published HIV-1 sequences of various subtypes downloaded from Los Alamos HIV Sequence Database (http://www.hiv.lanl.gov (accessed on 18 September 2022)). Sequencing reactions were performed at Azenta Life Sciences (formerly Genewiz, Chelmsford, MA, USA) on an Applied Biosystems 3730XL DNA Analyzer (Thermo Fisher Scientific 3730XL).

### 2.4. Sequence Alignment

Chromatograms resulting from sequencing reactions of the same amplicon were de novo assembled using Geneious Prime software v2021.2.2 (Biomatters, Auckland, New Zealand), and manual examination of the assemblies was performed to resolve ambiguous consensus base calls where possible. The amplicon was not included in further analysis if multiple peaks were present in chromatograms indicating more than one starting template in the PCR and/or if a sequencing primer failed to produce adequate useable sequence to complete de novo assembly. Consensus sequences generated from finished assemblies were aligned using MAFFT v7.450 [38] implemented in Geneious Prime, followed by manual alignment optimization. Due to a large number of insertions and deletions that are typically problematic to align and may bias phylogenetic analysis, hyper-variable regions in env (V1, V2 and V4 domains) were excluded. Consensus sequences that contained truncations >200 bp were removed from further analysis. Sequences were tested for the presence of hyper-mutations using the HYPERMUTE tool (http://www.lanl.gov (accessed on 5 March 2022)); sequences with a *p*-value of <0.01 were removed from the alignments. HIV-1 subtype was determined for consensus sequences using COMET via the online interface [39]. Average pairwise genetic distances for env and nef sequences taken at baseline and post-treatment from tumor DNA were calculated using the tn93 program (https://github.com/veg/tn93 (accessed on 4 April 2022)) and the Wilcoxon Rank Sum test was used to determine significant differences between groups. Compartmentalization in tissues was assessed using the tree topology-based structured Slatkin-Maddison test [40], implemented in HYPHY [41]. For each subject, evidence of compartmentalization was assessed between the plasma, PBMC, and tumor baseline sequences, between post-treatment PBMC and tumor sequences, and between baseline and post-treatment tumor sequences. Sequences were submitted to GenBank (Accession numbers OP272684-OP272861).

### 2.5. Phylogenetic Analysis

A preliminary maximum-likelihood phylogeny was estimated using sequences from all participants to ensure no cross-contamination of subjects was present, and individual env and nef maximum-likelihood (ML) phylogenies for each patient were estimated using IQ-TREE2 [42] under a general-time-reversible (GTR) nucleotide substitution model and gammap-distributed rate variation among sites. Statistical support of branches was assessed with 1000 bootstrap replicates. Phylogenies were visualized and midpoint rooted using FigTree software (https://github.com/rambaut/figtree, accessed on 2 February 2022).

### 2.6. Immunohistochemistry (IHC)

Serial sections of FFPE tissues from all the cases were used to evaluate HHV-8, CD68, and HIV-1 Nef protein by single-color IHC. IHC was performed on 4 μm FFPE tissue sections using a Leica Bond III platform with their Bond Polymer Refine detection system and associated reagents supplied by Leica Biosystems (Wetzlar, Germany). The processing of slide-mounted tissues was performed using the manufacturer conditions. Epitope Retrieval and antibody dilution were carried out according to the antibody: HHV-8 LANA (Cell Marque, clone: 13B10) 1:200, CD68 (Dako, clone: PG-M1) 1:100, and HIV-1 Nef (Santa Cruz Biotechnology, Inc., Santa Cruz, CA, USA) 1:50. The epitope retrieval for HHV-8 LANA and HIV-1 Nef was treated with Leica’s BOND Epitope Retrieval Solution 1 (citrate-based) at pH 6.0 for 20 min at 100 °C. For CD68, the epitope retrieval used Leica’s BOND Epitope Retrieval Solution 2 (EDTA based) at pH 9.0 for 20 min at 100 °C. Leica Antibody Diluent (Leica Biosystems) was used for the dilutions of all three mouse monoclonal antibodies. All primary antibodies were applied to the section for 15 min at room temperature. Primary antibody binding to tissue sections was visualized using Leica BOND Polymer Refine Detection: the Leica Refine Detection polymer was incubated for 8 min at room temperature, and DAB was incubated for 10 min at room temperature and counterstained with hematoxylin for 7 min at room temperature. The IHC images were acquired using Leica Versa 200 Automated Slide Scanner (Leica Biosystems).

## 3. Results

### 3.1. Clinical Characteristics of the Study Cohort and Sample Integrity

HIV-1 plasma viral load and CD4+ T cell counts were evaluated 6–14 days prior to ART initiation and are provided along with the demographics of the study participants (Table 1). Tumor biopsies were preserved in RNA-later and frozen at −80 °C until nucleic acid extraction (Supplemental Table S1). The RNA derived from all samples was of medium to high quality based on the Bioanalyzer RNA integrity number (RIN) values (Table 2)**.**

### 3.2. HIV DNA and RNA Sequences Were Obtained from KS Tumors at Baseline and Post-Treatment

Using samples obtained prior to ART initiation (baseline), HIV sequences were obtained from the plasma, PBMC DNA, tumor DNA, and tumor RNA for all four participants (Table 3). Amplification and sequencing primers are listed in Supplemental Table S2. After ~180–280 days of ART therapy (post-treatment), HIV DNA from tumors was sequenced from all four participants, while viral RNA from tumors (either spliced or unspliced) was sequenced in three of four participants (K09 was the exception). Post-treatment, PBMC HIV-DNA was also sequenced in three of four participants (K12 was the exception). The absence of post-treatment RNA plasma sequences from all four subjects was consistent with undetectable viral loads.

### 3.3. Post-Treatment Viral Diversity Remained High despite a Significant Reduction in HIV DNA

**Figure 1:**
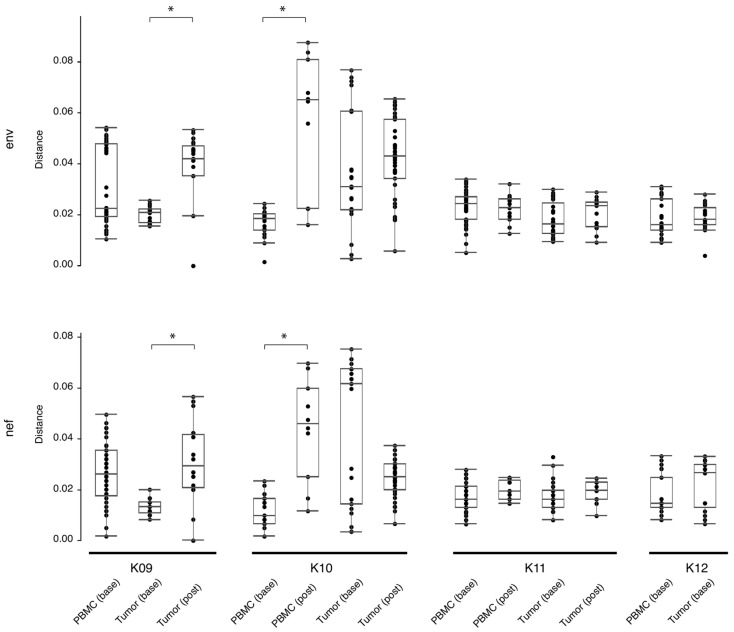
Estimates of evolutionary divergence between sequences from PBMCs and tumors, at baseline and post-treatment. Analyses were conducted using the Tamura-Nei 93 program (https://github.com/veg/tn93, accessed on 4 April 2022) and the Wilcoxon Rank Sum test applied to determine significance. Shown are the mean (black bar inside box), interquartile range (white box), and range (solid line terminating in black bars). Significance (*p* < 0.05) is indicated by a bracket and asterisk. Base = baseline, post = post-treatment, PBMC = peripheral blood mononuclear cells.

The number of HIV-DNA templates in KS tumors available for nested-PCR amplification was, on average, reduced 10-fold following ~180–280 days on ART (Table 4). At baseline, the average number of single-genome sequences (SGSs) per microgram of genomic tumor DNA was 5.61, while after ART therapy, the average number of SGSs per microgram was 0.57. While there was a reduction in the number of sequences derived from HIV DNA, in the three subjects with >1 post-treatment tumor sequence, tumor diversity measured with pairwise genetic distances between sequences was either unchanged (K10 and K11) or significantly increased (K09; *p* < 0.05) with respect to baseline diversity (Figure 1). Similarly, in the two subjects with >1 post-treatment PBMC sequence, post-treatment diversity was also either unchanged (K11) or significantly increased (K10; *p* < 0.05).

### 3.4. Completely Spliced RNA Transcripts Were Amplified More Frequently Post-Treatment

**Figure 2:**
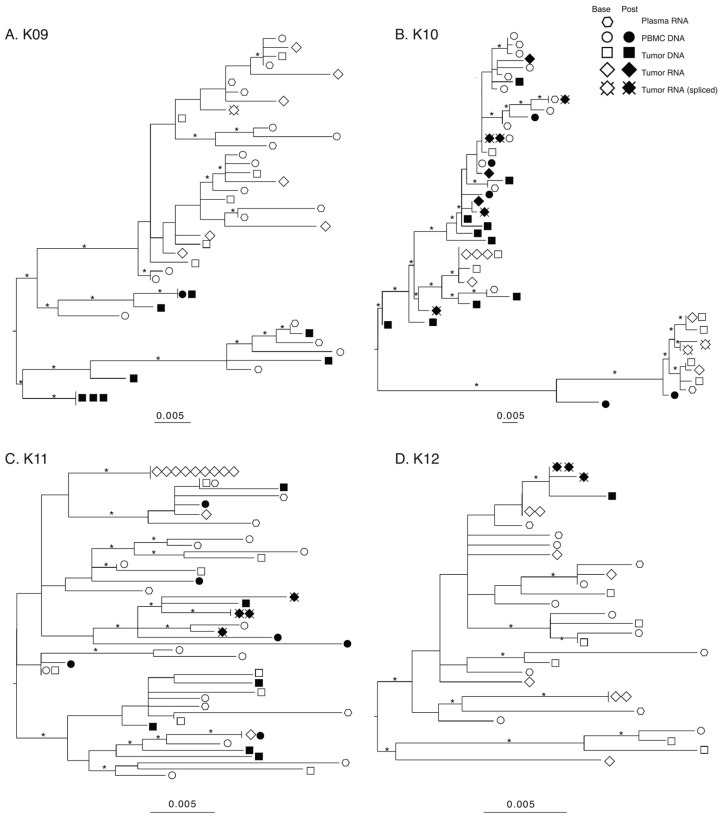
Maximum-likelihood (ML) phylogenies of *nef* sequences. Phylogenies for each patient were estimated using IQ-TREE2 under a general-time-reversible (GTR) nucleotide substitution model and gamma-distributed rate variation among sites. Statistical support of branches was assessed with 1000 bootstrap replicates and branches with 75% or more support are denoted with an asterisk. Phylogenies were visualized and midpoint rooted using FigTree.

The products produced during nested PCR of cDNA generated from tumor RNA form two different groups based on length. The longer products that were around 3.5 Kb originated from either HIV full-length genomic RNA or the rare partially spliced *vif* transcripts [43,44], while the shorter products, between 1.0 and 1.3 Kb, are from completely spliced HIV RNA transcripts. Sequencing the products revealed that at baseline, HIV RNA in tumors generated *env-nef* sequences, while in post-treatment tumors, spliced sequences were found almost exclusively (Table 3). As the shorter sequences from completely spliced HIV RNA did not contain the *env* sequence found in the longer products, these shorter sequences are shown only in the *nef* phylogenies as “Tumor RNA spliced” (Figure 2).

### 3.5. Lack of Compartmentalization and Maintenance of HIV Genetic Diversity in KS Tumors after 180–280 Days of ART

A preliminary maximum-likelihood (ML) phylogeny of all sequences confirmed a distinct population of HIV sequences for each patient, indicating no laboratory cross-contamination (Supplemental Figure S1)**.** The individual *env* and *nef* ML phylogenies for all four subjects are shown in *env* Supplemental Figure S2, *nef* Figure 2. The number of sequences included or removed from analysis can be found in Supplemental Figure S3; reasons for removal included hypermutation, truncation, double peaks in chromatograms indicating multiple templates present, and sequencing primer failure.

Several patterns are consistent among all subjects/genes. First, sequences from baseline plasma, PBMC, and tumor were interspersed on all trees, indicating a lack of compartmentalization. Second, baseline tumor RNA sequences are often more closely related with plasma or PBMC sequences rather than a tumor DNA sequence, suggesting an even broader underlying spectrum of genetic diversity. Third, the post-treatment tumor HIV-DNA sequences were derived from at least two different well-supported clades for the three subjects with >1 sequence, suggesting maintenance of genetic diversity. Fourth, only one subject (K09) showed identical post-treatment DNA sequences. This further confirms the underlying extent of genetic diversity in the post-treatment tumor environment. Finally, in two of the three subjects with post-treatment spliced tumor RNA sequences (K10 and K11), the spliced sequences are found on multiple well-supported branches of their *nef* phylogenies. Furthermore, in both subjects, at least one of the post-treatment spliced tumor RNA sequences was more closely related to a baseline plasma sequence rather than a tumor sequence. These observations suggest that even beyond the maintenance of HIV-DNA diversity in the tumor post-treatment, the expression of viral transcripts originates from a diverse HIV-DNA population as well.

To confirm the observation of a lack of population structure among tissues and at baseline and post-treatment, we performed the structured Slatkin–Maddison test for compartmentalization. The only significant structure was found for K11 among baseline tissues for *env*, confirming the pattern of panmixia (Supplemental Table S3).

### 3.6. HIV Nef Protein Persists in CD68+ Macrophages after ART

**Figure 3:**
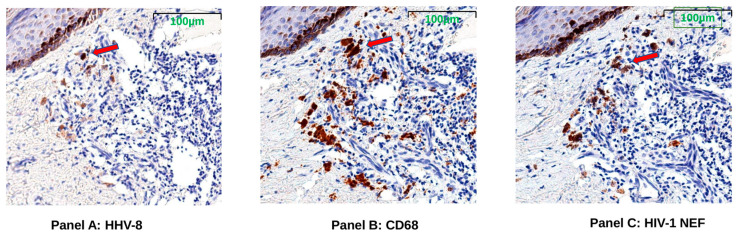
Representative images (K10) showing HHV-8 LANA (Panel **A**), CD68 (Panel **B**), and HIV-1 Nef proteins (Panel **C**). Sequential tissue sections were taken from post-ART treatment Kaposi’s Sarcoma (KS) skin tumor biopsy of subject K10 for imaging with single-color immunohistochemistry (IHC) staining. Brown color indicates antibody binds to specific protein. Red arrows indicate a representative area where HIV-1 Nef and CD68, a tumor-associated macrophage marker, localized in the HHV-8-positive KS tumor region just under the margin of the epidermis (darkly pigmented cells near top left of images).

To test whether the spliced RNAs encoding nef were translated in the KS tumor tissue, stains for HIV-1 Nef, KS tumor marker HHV-8 latent nuclear antigen (LANA), and macrophage marker CD68 proteins were evaluated in serial sections of FFPE tissues. The presence of HIV-Nef protein was observed in all KS biopsy tissues from both pre-ART and post-ART treatment cases by single-color IHC staining. Representative tissue images from serial sections taken from subject K10 demonstrate HHV-8 positivity below the epidermal layer; HIV-1 Nef protein appears in cells with the same shape and localization of CD68+ macrophages (Figure 3).

## 4. Discussion

In this study, we explored whether KS tumors can serve as reservoirs of HIV diversity and activity after plasma viral suppression was achieved on ART. We analyzed HIV env and nef sequences from plasma, PBMCs, and tumor biopsies before ART initiation (baseline) and then in PBMCs and tumors after ~180–280 days of ART (post-treatment). The subjects were confirmed to have achieved a sustained plasma viral load below the limit of detection and had rebounding CD4+ T-cell counts following ART. While ART had succeeded in these metrics, these subjects still exhibited cutaneous KS lesions post-treatment. Our results show that in subjects with persistent KS after ~180–280 days of ART, HIV-DNA diversity was maintained, spliced RNA transcripts continued to be produced, and HIV Nef protein appears localized to CD68+ macrophages in KS skin tumors. These results indicate that HIV located in KS tumors continues to be active and shapes the tumor microenvironment, even after ART has reduced plasma HIV viral load to undetectable levels and restored immune function.

HIV RNA and/or DNA was amplified from all tumor biopsies in the study, demonstrating that cancer tissues contain substantial amounts of HIV, a result similar to previous studies [10,26,27,28,29,30]. Furthermore, while the number of HIV sequences obtained from each microgram of genomic DNA was reduced 10-fold post-treatment, HIV env and nef sequence populations from the tumor tissues in this study before and after ART had similar or increased ranges of pairwise genetic distances (Figure 1). This finding indicates a preservation of genetic diversity in the tumor niche following ~180–280 days of treatment.

A recent study on HIV viral dynamics in Uganda highlighted the influence of different subtypes in creating a diverse reservoir for subjects on ART [45]. They hypothesized that the smaller, but more diverse, peripheral HIV-1 reservoir in Ugandan subjects might be associated with viral (e.g., subtype, pathogenicity) and/or host (e.g., co-infections or co-morbidities) factors contributing to less clonal expansion than found in those infected with HIV-1 subtype B. In our four subjects, three were subtype A1, while one (K10) was subtype D (Table 3). Future studies will examine a larger cohort to fully assess the impact of subtype on diversity.

Only one subject (K09, post-treatment) has potential evidence of clonal expansion of infected cells, indicated by some identical env and nef DNA sequences (three identical tumor DNA sequences, one identical sequence in both tumor DNA and PBMC DNA). Characterization of the integration sites would be necessary to confirm clonal expansion in this subject. Via use of the CPS tool [46], which calculates the probability of subgenomic sequences to correctly detect clonality, our PCR primers have an ~80% probability (CPS Score 81.333, st. dev. 27.244) to correctly predict that these identical sequences arose from a clonally expanding cell. While clonal expansion plays a key role in the maintenance of viral reservoirs (reviewed in [47,48]), it was not a dominant factor influencing HIV genetic diversity in KS tumors of our subjects on ART for ~180–280 days.

We amplified a similar number of sequences from K10 and K11 at baseline and post-treatment and obtained similar phylogenies. The lineages that remain after treatment are distributed throughout the phylogeny, without obvious compartmentalization between the blood and tumor samples. In our previous study of HIV-1 intrahost population structure in skin and visceral KS tumors and non-tumor tissues at autopsy [10], two-thirds of the subjects had evidence of compartmentalization in tumor vs. nontumor virus; however, some limited mixing did occur. The specimens in this study were from living subjects, where we would expect that punch biopsies from highly vascularized skin tumors would contain a substantial amount of blood. The longitudinal biopsies were also not necessarily from the same tumor. These two factors likely mask compartmentalized blood vs. tumor virus subpopulations, if they were, in fact, present.

HIV-1 viral gene expression follows a strict chronological order [49,50,51]. HIV RNA generated inside an infected cell includes unspliced full-length genomic RNA and partially or completely spliced RNA transcripts with specific splice acceptor and donor sites that correspond to various HIV proteins (reviewed in [52,53]). The second-round PCR forward primer is located just upstream of the acceptor 5 (A5) splice sites utilized in nef transcripts. Given this primer placement, the distinction between the exact gene represented by the spliced transcripts is masked. These sequences are believed to arise from completely spliced transcripts, given that they contain a junction using the splice acceptor 7 site, which is found in all completely spliced tat/rev/nef transcript conformations [54].

Tumor HIV-RNA single-genome sequences (SGSs) were generated in all baseline tumor samples and in three of four post-treatment biopsies, where K09 was the lone exception. Prior to ART, most RNA sequences were unspliced (Table 3). At baseline, completely spliced RNA transcripts were found in K09 (1/7 SGS) and K10 (2/6 SGS), but no completely spliced RNA transcripts were among the sequences from K11 (11 SGS) and K12 (6 SGS). The opposite pattern was observed post-treatment: K09 had no SGSs from tumor RNA, K10 had five completely spliced transcripts and three *env-nef* SGS, K11 only had four completely spliced transcripts, and K12 had three completely spliced transcripts.

Typically, after treatment, a more severe reduction in completely spliced RNA transcripts compared to unspliced transcripts is found in PBMCs [55,56,57,58,59] and tissues [60,61,62]. While HIV-DNA sequences deriving from post-treatment PBMC DNA were found in three of four subjects, no RNA sequences were obtained, even after two separate cell aliquots (each ~1 × 10^6^) were assayed. The persistence of fully spliced RNA in most of the post-treatment tumor biopsies and the absence of HIV RNA in PBMCs might be due to different mechanisms that govern HIV expression in tissues [61,63]. However, one recent study [60] found that completely spliced HIV transcripts were usually detected in PBMCs but only rarely in tissues. Our data indicate that the HIV-infected cells in the KS tumor niche usually maintain the ability to produce completely spliced viral transcripts necessary to produce viral proteins (Tat/Rev/Nef) after ~180–280 days of ART.

Immunohistochemistry (IHC) staining supports the continued presence of HIV Nef in post-treatment tumor biopsies. The HIV Nef appears to be localized to CD68+ tissue macrophages in the KS tumors. While the precise role that HIV Nef might play in driving KS persistence post-ART is currently unknown, the identification of HIV Nef protein in tumor tissue macrophages suggests a mechanism of pathogenic persistence that warrants further investigation. HIV-infected macrophages have played major roles in either pathogenesis or viral persistence in both HIV-associated dementia and HIV-associated large-cell lymphoma [64,65]. Macrophages as sites of disease-associated HIV integration were first shown by Mack et al. when both lymphoma and dementia tissue-insertion sites were mapped near activation genes, a process known to neutralize macrophage’s apoptosis signal, thus, allowing these cells to persist [66].

HIV Nef protein has been found in plasma [67], extracellular vesicles [68,69,70], PBMCs [71,72], and lungs [73] of aviremic subjects on ART. A recent study [74] found that HIV Nef specific T-cell responses are highly stable on long-term ART, very significantly associated with measures of the viral reservoir (e.g., HIV DNA and HIV cell-associated RNA) and indicative of recent T-cell recognition of Nef antigens. While the robust T-cell immune responses to Nef should facilitate the clearance of Nef-producing cells, the immunosuppressive functions of Nef through selective modification of MHC 1 presentation to avoid CTL and NK cell killing allows HIV-infected cells expressing Nef to persist. Nef also influences several different cellular pathways to inhibit [75,76,77] or promote [78,79] apoptosis. Considering the continued production of Nef by infected cells on ART and modulation of immune and apoptotic pathways by Nef, several therapies have been proposed to eliminate Nef-producing cells and reduce the viral reservoir [80,81,82,83]. Given that continued nef expression and Nef protein production is localized in KS tumors during ART, the use of these therapies to ameliorate KS should be investigated.

The role of HIV Nef in KS tumor maintenance and metastasis should be further explored. One limitation of this study is the small number of subjects, and a thorough investigation of a larger cohort is required for statistical support for our findings. The study subjects received outdated ART regimens during the original ARKS trial and future studies would be needed with updated standard regimens, including dolutegravir. We were also limited by PCR primer placement in our ability to identify exactly which protein was ultimately produced by the completely spliced transcripts we found. A more in-depth exploration of the kinds and frequencies of the HIV transcripts that persist in KS tumors during ART therapy is underway. While our methods sequence about half the HIV genome, full-genome sequencing could further elucidate the role that defective genomes play in KS tumors, and integration site analysis would detail clonal expansion of infected cells. The future use of single-cell assays will further define exactly which cells are producing HIV transcripts and proteins, although the limited size of punch biopsies might present challenges.

Our biopsy IHC staining revealed the continued presence of Nef in KS tumors; however, analysis of plasma and circulating extracellular vesicles for Nef will establish if Nef is a biomarker of KS persistence in ART [67,84]. CD68-specific antibody was used to stain tumor-associated macrophages in the KS tumor microarrays [85,86]. However, co-stains with other antibodies will need to be performed to further characterize these CD68+ cells and address the possibility of CD68 antibody binding to targets other than macrophages [85] or to elucidate the M1/M2 activation state [11,12]. Future studies could also include markers for T-cell subsets and other HIV proteins, such as HIV Tat, which has been shown to play a role in KS [86,87], to further characterize the interplay between the cells of the tumor microenvironment and ongoing HIV expression during ART.

In summary, this study shows that in subjects with persistent KS after ~180–280 days of ART, HIV diversity is maintained, RNA transcripts are expressed and spliced, and HIV Nef protein is localized to CD68+ macrophages in KS skin tumors. These results indicate that HIV located in KS tumors continues to be transcriptionally and translationally active, potentially influencing the tumor microenvironment even after ART has reduced plasma HIV viral load to undetectable levels and restored immune function. Continued studies using additional methods that define the HIV transcriptionally and translationally active reservoir in the KS tumor niche could lead to novel therapies for PLWH with KS.

## Figures and Tables

**Table 1:** The demographics of study subjects.

Subject ID	Gender	Age (Years)	Days of Pre-Randomization	HIV Load (Copies/mL)	CD4 Cells Count (Cells/mm^3^)
K09	Male	44	11	556,826	124
K10	Male	38	6	39,996	113
K11	Female	23	12	479,742	142
K12	Female	37	14	272,745	624

**Table 2:** Tumor biopsy nucleic acid yield and RNA quality.

		***Baseline Tumors***					
**ID**	**ACSR ID**	**Biopsy (mg)**	**DNA Conc (ng/µL)**	**Total DNA (ng)**	**RNA Conc (ng/µL)**	**Total RNA (ng)**	RIN *
K09	KPA5178	21.8	93.8	37,520	57.6	5760	5.7
K10	KPA5552	34.5	250	100,000	224	22,400	7.0
K11	KPA5763	21.6	80.4	32,160	71.2	7120	5.1
K12	KPA6014	24.7	177	70,800	90.4	9040	7.2
averages		26.3	158.3	63,306.7	101.7	10,173.3	6.6
		***Post-Treatment Tumors***					
**ID**	**ACSR ID**	**Biopsy (mg)**	**DNA Conc (ng/µL)**	**Total DNA (ng)**	**RNA Conc (ng/µL)**	**Total RNA (ng)**	RIN *
K09	KPA5178	30.1	184	73,600	182.0	18,200	9.3
K10	KPA5552	27.4	169	67,600	212.2	21,224	8.7
K11	KPA5763	21.6	122	48,800	140.6	14,056	7.2
K12	KPA6014	18.9	61.6	24,640	91.8	9184	7.6
averages		23.0	119.7	47,866.7	132.1	13,206.7	7.4

* RNA integrity numbers (RIN) are calculated by Agilent Bioanalyzer software B.02.11.

**Table 3:** HIV subtype and number of sequences, excluding truncated, incomplete, or hypermutated sequences.

Subject ID	ACSR ID	COMET Subtype	Timepoint	Plasma	PBMC	Tumor	Tumor
				RNA	DNA	DNA	RNA *
K09	KPA5178	A1	baseline	10	9	6	7 (1)
			post-treatment	0	1	8	0
K10	KPA5552	A1	baseline	7	7	7	6 (2)
			post-treatment	0	5	10	3 (5)
K11	KPA5763	D	baseline	8	10	8	11
			post-treatment	0	6	6	0 (4)
K12	KPA6014	A1	baseline	6	7	8	6
			post-treatment	0	0	1	0 (3)

* Number of spliced RNA transcripts is in parenthesis.

**Table 4:** Calculated number of *env-nef* sequences per microgram tumor DNA.

***Baseline Tumor***				
**BIE**	**ACSR**	SGS *	**µg DNA Used for PCR**	**SGS/µg DNA**
K09	KPA5178	6	0.49	12.30
K10	KPA5552	7	1.30	5.38
K11	KPA5763	8	1.58	5.08
K12	KPA6014	6	3.47	1.73
	averages	7.0	1.66	5.61
***Post-Treatment Tumor***				
**BIE**	**ACSR**	SGS *	**µg DNA Used for PCR**	**SGS/µg DNA**
K09	KPA5178	8	8.10	0.99
K10	KPA5552	10	7.10	1.41
K11	KPA5763	6	10.25	0.59
K12	KPA6014	1	5.17	0.19
	averages	4.3	7.64	0.57

* SGS = single genome sequence originating from single starting template.

## Data Availability

Sequences were submitted to GenBank (Accession numbers OP272684-OP272861).

## Supplementary Materials

The following supporting information can be downloaded at: https://www.mdpi.com/article/10.3390/v14122774/s1, Figure S1: Maximum-Likelihood phylogeny of all env sequences for 4 subjects, variable regions removed. Sequences for each subject are exclusive to a single main branch on the phylogeny, indicating no cross-contamination between subjects. Statistical support of branches was assessed with 1000 bootstrap replicates and branches with 100% support are denoted with an asterisk. Legend: K09 = square, K10 = triangle, K11 = circle, K12 = diamond; Figure S2: Maximum-likelihood (ML) phylogenies of env sequences. Phylogenies for each patient were estimated using IQ-TREE2 under a general-time-reversible (GTR) nucleotide substitution model and gamma-distributed rate variation among sites. Statistical support of branches was assessed with 1000 bootstrap replicates and branches with 75% or more support are denoted with an asterisk. Phylogenies were visualized and midpoint rooted using FigTree; Figure S3: Number of usable and unusable sequences at baseline and post-treatment timepoints. U—Usable sequences were complete *env-nef* HIV genome sections or correctly spliced RNA transcripts. MP—Manual examination of the chromatograms was used to determine if multiple peaks existed at each nucleotide. SPF—Sequencing primers failed to work for some products. H—Hypermutated sequences were determined by the Los Alamos HYPERMUTE tool. T—Truncated sequences; Table S1: Availability of baseline and post-treatment specimens for 4 subjects with the interval days between samples; Table S2: Primers used for SGS PCR and sequencing; Table S3: *p*-values for the structured Slatkin–Maddison test for compartmentalization.
