# Supplementary material for: The Persistence of HIV Diversity, Transcription, and Nef Protein in Kaposi’s Sarcoma Tumors during Antiretroviral Therapy
Source: Viruses. 2022 Dec 13;14(12):2774. doi: 10.3390/v14122774 (PMC9782636; doi:10.3390/v14122774)
Supplement: Supplementary file 1 [file viruses-14-02774-s001.zip › viruses-2068062-supplementary.pdf]

Table S1. Availability of baseline and post-treatment specimens for 4 subjects with the interval days between samples.

Subject ID	Specimen Type	Baseline	Post-ART treatment	Interval Days*
K09	Biopsy in RNA Later	Yes	Yes	294
	PBMC	Yes	Yes	292
	Plasma	Yes	Yes	292
	Biopsy FFPE block	N/A	Yes	39
K10	Biopsy in RNA Later	Yes	Yes	177
	PBMC	Yes	Yes	174
	Plasma	Yes	Yes	174
	Biopsy FFPE block	Yes	Yes	176
K11	Biopsy in RNA Later	Yes	Yes	179
	PBMC	Yes	Yes	180
	Plasma	Yes	Yes	180
	Biopsy FFPE block	N/A	Yes	192
K12	Biopsy in RNA Later	Yes	Yes	184
	PBMC	Yes	Yes	182
	Plasma	Yes	Yes	182
	Biopsy FFPE block	Yes	Yes	184

* The days of specimen collection between baseline and post-ART treatment or between HIV virus assay date and post-ART treatment if baseline specimen unavailable.

Table S2. Primers used for SGS PCR and sequencing.

Purpose	Primer	5' to 3' Sequence	HXB2 Position*	Reference
SGS	VIF_F2	TGGAAAGGTGAAGGGGCAGTA	4956–4976	[89]
First Round	3UTR_R1	TATTGAGGCTTAAGCAGTGGGTTC	9615–9592	[89]
SGS	ENV_F1	GGCTTAGGCATCTCCTATGGCAGGAAGAA	5954–5982	[89]
Second Round	3UTR_R6	GCTTATATGCAGCATCTGAGGG	9497–9518	[89]
Sequencing Primers	ENV_F2	CATGCCTGTGTACCCACAGAC	6438–6458	this study
	ENV_F3	CCAATTCCYATACATTATTGTGC	6858–6880	this study
	120_F2	AGACCTGGAGGAGGARATATGA	7629–7650	this study
	120_R3	CGCCCATMGTGCTTCCTGCTG	7818–7838	this study
	NEF_F1	CAGCAGGAAGCACTATGGGCG	7798–7818	[89]
	NEF_F4	ATGATAGTAGGAGGCTTMATAGG	8283–8305	this study
	NEF_R6	GGTAGCTGAAGAGGCACAG	8529–8511	this study

*base pair positions relative to the HIV-1 HXB2 reference

Table S3. p-values for the structured Slatkin–Maddison test for compartmentalization.

Patient	Gene	TM base vs TM post	TM post vs PBMC post	TM base vs PL base vs PBMC base
K09	*env*	0.078	0.549	0.738
K09	*nef*	0.204	0.935	0.607
K10	*env*	1	0.774	0.011*
K10	*nef*	1	0.538	0.022
K11	*env*	0.636	0.045	0.289

K11	*nef*	1	0.590	0.547
K12	*env*	1	0.989	0.996
K12	*nef*	1	0.896	0.973

Baseline=base, post-treatment=post, TM=tumor, PL=plasma, PBMC=peripheral mononuclear cells.

* Indicates p-value was significantly lower than the alpha level of p=0.002, which accounts for multiple tests.

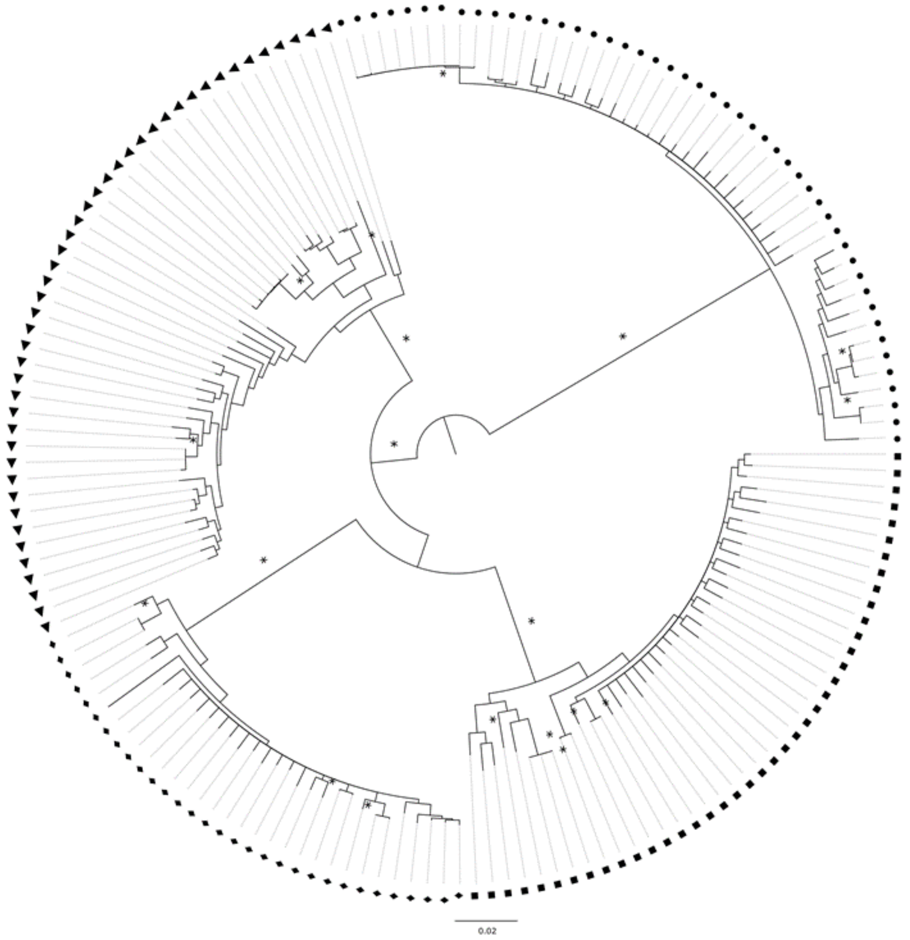

Figure S1 Maximum-Likelihood phylogeny of all env sequences for 4 subjects, variable regions removed. Sequences for each subject are exclusive to a single main branch on the phylogeny, indicating no cross-contamination between subjects. Statistical support of branches was assessed with 1000 bootstrap replicates and branches with 100% support are denoted with an asterisk. Legend: K09=square, K10=triangle, K11=circle, K12=diamond.

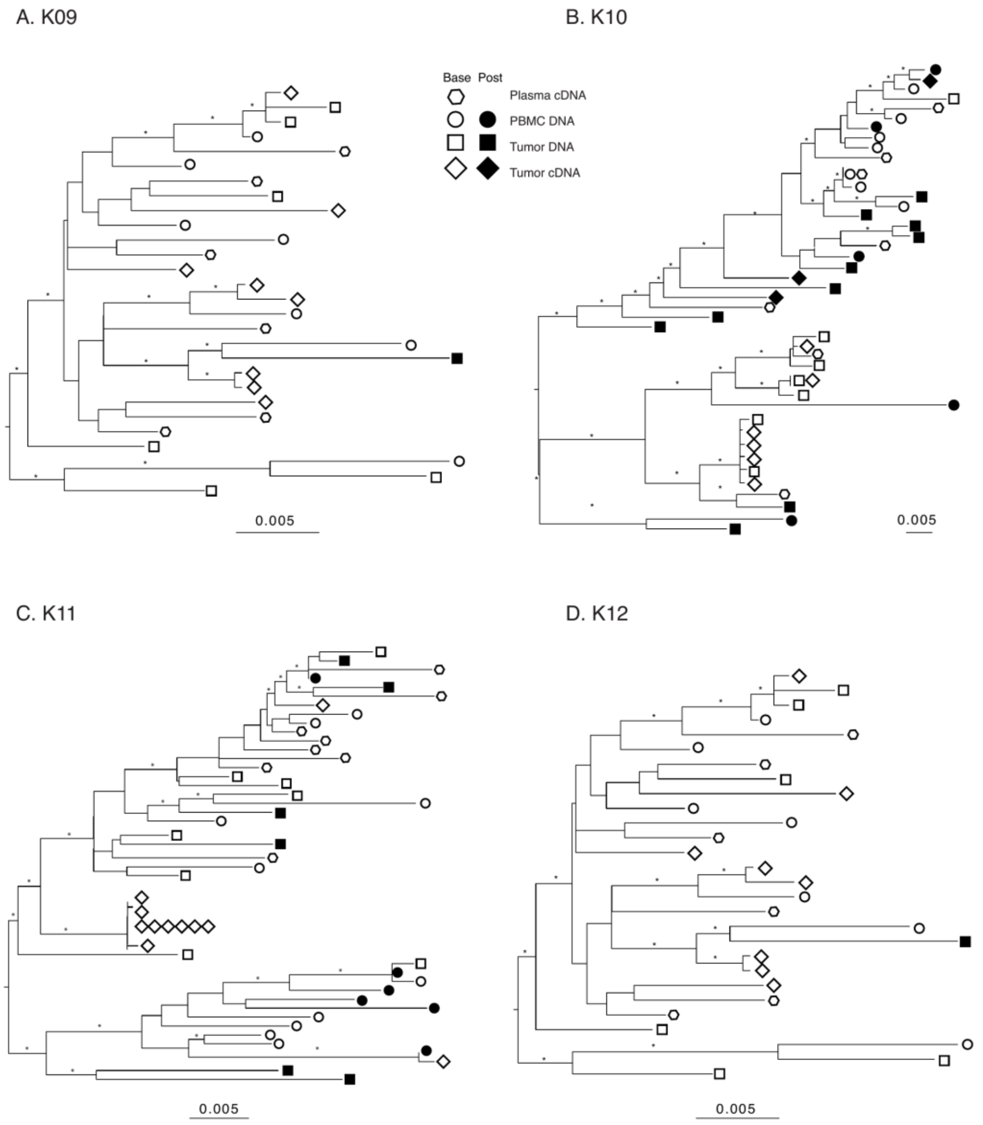

Figure S2. Maximum-likelihood (ML) phylogenies of *env* sequences. Phylogenies for each patient were estimated using IQ-TREE2 [94] under a general-time-reversible (GTR) nucleotide substitution model and gamma-distributed rate variation among sites. Statistical support of branches was assessed with 1000 bootstrap replicates and branches with 75% or more support are denoted with an asterisk. Phylogenies were visualized and midpoint rooted using FigTree.

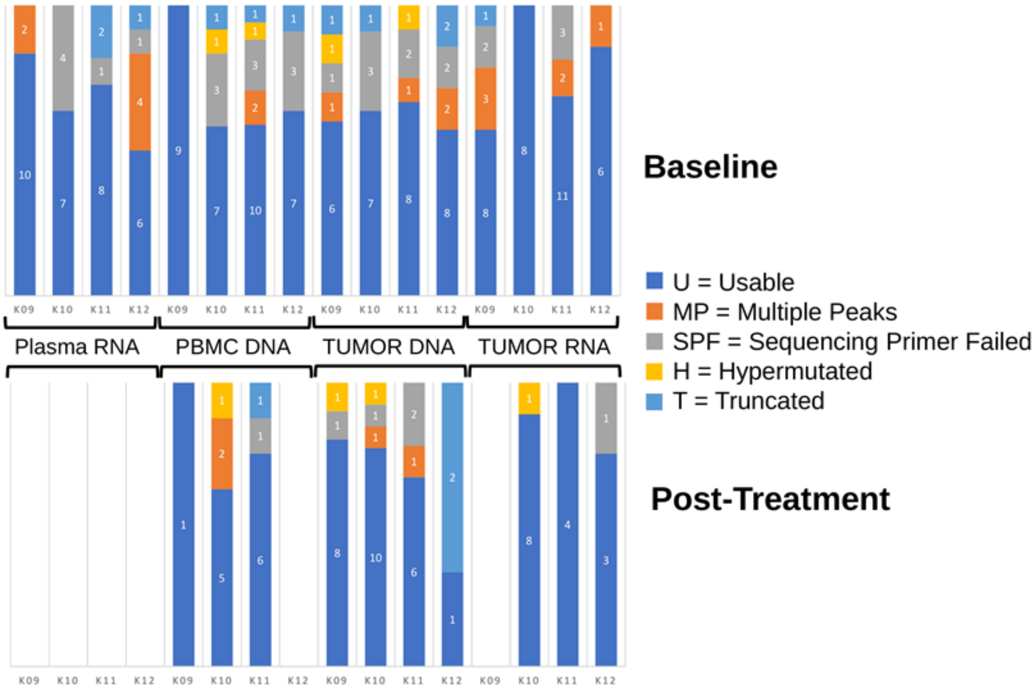

Figure S3. Number of usable and unusable sequences at baseline and post-treatment timepoints. U—Usable sequences were complete *env-nef* HIV genome sections or correctly spliced RNA transcripts. MP—Manual examination of the chromatograms was used to determine if multiple peaks existed at each nucleotide. SPF—Sequencing primers failed to work for some products. H—Hypermutated sequences were determined by the Los Alamos HYPERMUTE tool. T—Truncated sequences.
